# Marker-assisted selection strategy to pyramid two or more QTLs for quantitative trait-grain yield under drought

**DOI:** 10.1186/s12284-018-0227-0

**Published:** 2018-05-29

**Authors:** Arvind Kumar, Nitika Sandhu, Shalabh Dixit, Shailesh Yadav, B. P. M. Swamy, Noraziyah Abd Aziz Shamsudin

**Affiliations:** 1 International Rice Research Institute, DAPO Box 7777, Metro Manila, Philippines; 2 Current address: Faculty of Science and Technology, Universiti Kebangsaan Malaysia, 43600 Bangi, Selangor Malaysia

**Keywords:** Drought, Drought yield QTLs, Marker-assisted selection breeding strategy, Pyramiding, Rice

## Abstract

**Background:**

Marker-assisted breeding will move forward from introgressing single/multiple genes governing a single trait to multiple genes governing multiple traits to combat emerging biotic and abiotic stresses related to climate change and to enhance rice productivity. MAS will need to address concerns about the population size needed to introgress together more than two genes/QTLs. In the present study, grain yield and genotypic data from different generations (F_3_ to F_8_) for five marker-assisted breeding programs were analyzed to understand the effectiveness of synergistic effect of phenotyping and genotyping in early generations on selection of better progenies.

**Results:**

Based on class analysis of the QTL combinations, the identified superior QTL classes in F_3_/BC_1_F_3_/BC_2_F_3_ generations with positive QTL x QTL and QTL x background interactions that were captured through phenotyping maintained its superiority in yield under non-stress (NS) and reproductive-stage drought stress (RS) across advanced generations in all five studies. The marker-assisted selection breeding strategy combining both genotyping and phenotyping in early generation significantly reduced the number of genotypes to be carried forward. The strategy presented in this study providing genotyping and phenotyping cost savings of 25–68% compared with the traditional marker-assisted selection approach. The QTL classes, *Sub1 + qDTY*_*1.1*_ *+ qDTY*_*2.1*_ + *qDTY*_*3.1*_ and *Sub1 + qDTY*_*2.1*_ + *qDTY*_*3.1*_ in Swarna-Sub1, *Sub1* + *qDTY*_*1.1*_ + *qDTY*_*1.2*_, *Sub1* + *qDTY*_*1.1*_ + *qDTY*_*2.2*_ and *Sub1 + qDTY*_*2.2*_ + *qDTY*_*12.1*_ in IR64-Sub1, *qDTY*_*2.2*_ + *qDTY*_*4.1*_ in Samba Mahsuri, *Sub1* + *qDTY*_*3.1*_ + *qDTY*_*6.1*_ + *qDTY*_*6.2*_ and *Sub1* + *qDTY*_*6.1*_ + *qDTY*_*6.2*_ in TDK1-Sub1 and *qDTY*_*12.1*_ + *qDTY*_*3.1*_ and *qDTY*_*2.2*_ + *qDTY*_*3.1*_ in MR219 had shown better and consistent performance under NS and RS across generations over other QTL classes.

**Conclusion:**

“Deployment of this procedure will save time and resources and will allow breeders to focus and advance only germplasm with high probability of improved performance. The identification of superior QTL classes and capture of positive QTL x QTL and QTL x background interactions in early generation and their consistent performance in subsequent generations across five backgrounds supports the efficacy of a combined MAS breeding strategy”.

**Electronic supplementary material:**

The online version of this article (10.1186/s12284-018-0227-0) contains supplementary material, which is available to authorized users.

## Background

Rice breeding methodology followed in the past as well as the present ranges from conventional breeding (Singh et al. 1998; Xinglai et al. 2006; Baenziger et al. 2008; Obert et al. 2008; Brick et al. 2008; Kumar et al. 2014), hybrid breeding (Shull 1948; Reif et al. 2005), marker-assisted breeding (MAB; Price 2006; McNally et al. 2009; Breseghello and Sorrells 2006; Kumar et al. 2014), and transgenic breeding (Bhatnagar-Mathur et al. 2008; Yang et al. 2010) to genome-wide association studies and genomic selection (Brachi et al. 2012; Huang et al. 2010; Begum et al. 2015; Biscarini et al. 2016). Grain yield as well as resistance against existing as well as emerging biotic and abiotic stresses is not a straightforward result of understanding the physiological, biochemical, and molecular mechanisms of genetic loci. Three major interactions, i) interaction between genes for the same trait, ii) genes for different traits, and iii) interactions of genes with environments and genetic background restricting the use of QTLs in introgression programs (Kumar et al. 2014; Wang et al. 2012; Xue et al. 2009; Almeida et al. 2013; Elangovan et al. 2008; Cuthbert et al. 2008; Heidari et al. 2011; Bennett et al. 2012). Selection of an appropriate donor/recipient to create desirable variability (Mondal et al. 2016; Dixit et al. 2014) and precise selection under variable conditions, environments, and stress intensity levels is must. A large population size is generally required for selecting appropriate plants possessing the needed gene combinations, desired plant type, and higher yield. An integration of modern, novel, and affordable breeding strategies with knowledge of associated mechanisms, interactions, and associations among related or unrelated traits/factors is necessary in rice breeding improvement programs.

The conventional breeding approach involving a series of phenotyping and genotyping screening of a large population to obtain desired variability and a high frequency of favorable genes in combination was earlier followed by several drought breeding program (Kumar et al. 2014). A conventional breeding approach involving sequential selection of large segregating populations for biotic (bacterial late blight, blast) and abiotic stresses (drought, submergence) across generations helped breeders to develop breeding lines combining tolerance of both stresses. Superior lines in terms of acceptable plant type, grain yield, and quality traits and stable performance under different environments are promoted for release (Kumar et al. 2014; Sandhu and Kumar 2017).

Modern molecular breeding strategies have been implemented to practice a more precise, quick and cost-effective breeding strategy compared to traditional conventional rice breeding improvement programs. Previously, many QTLs for grain yield under drought using different strategies such as selective/whole-genome genotyping, bulk segregant analysis (Vikram et al. 2011; Yadaw et al. 2013; Mishra et al. 2013; Sandhu et al. 2014; Ghimire et al. 2012) have been identified. The successful introgression and pyramiding of the identified genetic regions in different genetic backgrounds using marker-assisted backcrossing (Yadaw et al. 2013; Mishra et al. 2013; Sandhu et al. 2014; Venuprasad et al. 2009; Sandhu et al. 2013; Sandhu et al. 2015) has been reported. Accurate repetitive phenotyping in multi-locations and multi-environments under variable growing conditions is required to evaluate the performance and adaptability of the developed MAB products. There have been several examples of introgression of single genes for both biotic and abiotic stresses (gall midge – Das and Rao 2015; blast – Miah et al. 2016; brown plant hopper – Jairin et al. 2009; submergence – Septiningsih et al. 2009) in the background of popular high-yielding varieties as well as introgression of more than one gene for biotic stresses (*xa5 + xa13 + Xa21* - Singh et al. 2001, Kottapalli et al. 2010; *Xa21 + xa13* - Singh et al. 2011) for oligogenic traits controlled by major genes.

Several major large-effect QTLs such as *qDTY*_*1.1*_ (Vikram et al. 2011; Ghimire et al. 2012), *qDTY*_*2.1*_ (Venuprasad et al. 2009), *qDTY*_*2.2*_ (Venuprasad et al. 2007; Swamy et al. 2013), *qDTY*_*3.1*_ (Venuprasad et al. 2009), *qDTY*_*4.1*_ (Swamy et al. 2013), *qDTY*_*6.1*_ (Venuprasad et al. 2012), *qDTY*_*10.1*_ (Swamy et al. 2013), and *qDTY*_*12.1*_ (Bernier et al. 2007) for grain yield under reproductive-stage (RS) drought stress have been identified. A total of 28 significant marker trait associations were detected for yield-related trait in genome wide association study of japonica rice under drought and non-stress conditions (Volante et al. 2017). Moreover, each of these identified QTLs has shown a yield advantage of 300–500 kg ha^− 1^ under RS drought stress depending upon the severity and timing of the drought occurrence. However, in order to provide farmers with an economic yield advantage under drought, it is necessary that two or more such QTLs be combined to obtain a targeted yield advantage of 1.0 t ha^− 1^ under severe RS drought stress (Sandhu and Kumar 2017; Kumar et al. 2014).

Polygenic traits governed by more than one gene within the identified QTLs do not follow the simple rule of single gene introgression. The positive/negative interactions of alleles within QTLs and with the genetic background (Dixit et al. 2012a, b), pleiotropic effect of genes and linkage drag (Xu and Crouch 2008; Vikram et al. 2015; Vikram et al. 2016; Bernier et al. 2007; Venuprasad et al. 2009; Vikram et al. 2011; Venuprasad et al. 2012) played an important role in determining the effect of introgressed loci. The reported linkage drag of the *qDTY* QTLs has been successfully broken and individual QTLs have been introgressed into improved genetic backgrounds (Vikram et al. 2015). To identify an appropriate number of plants with positive interactions and high phenotypic expression, MAB requires genotyping and phenotyping of large numbers of plants/progenies in each generation from F_2_ onwards. In this case, MAB for more than two genes/QTLs is not a cost-effective approach. The population size to be genotyped and phenotyped for complex traits such as drought increases significantly as two or more QTLs are considered for introgression. To enhance breeding capacity to develop climate-resilient rice cultivars, there is a strong need to develop a novel, cost/labor-effective, and high-throughput breeding strategy. The effective integration of molecular knowledge into breeding programs and making MAB cost-effective enough to be fully adapted by small- or moderate-sized breeding programs are still a challenge.

In the present study, we closely followed the marker-assisted introgression of two or more QTLs for RS drought stress in the background of rice varieties; Swarna-Sub1, IR64-Sub1, Samba Mahsuri, TDK1-Sub1, and MR219 from F_3_ to F_6_/F_7_/F_8_ generations. Class analysis for different combinations of QTLs for yield under RS drought stress as well as under irrigated control conditions was performed with the aim to understand the effectiveness of synergistic effect of phenotyping and genotyping in early generations on selection of better progenies. We hypothesized that a QTL class that has performed well in an early generation may maintain its performance across generations/years and seasons.

## Results

### Performance of lines introgressed with QTLs for grain yield under drought

**Fig. 1:**
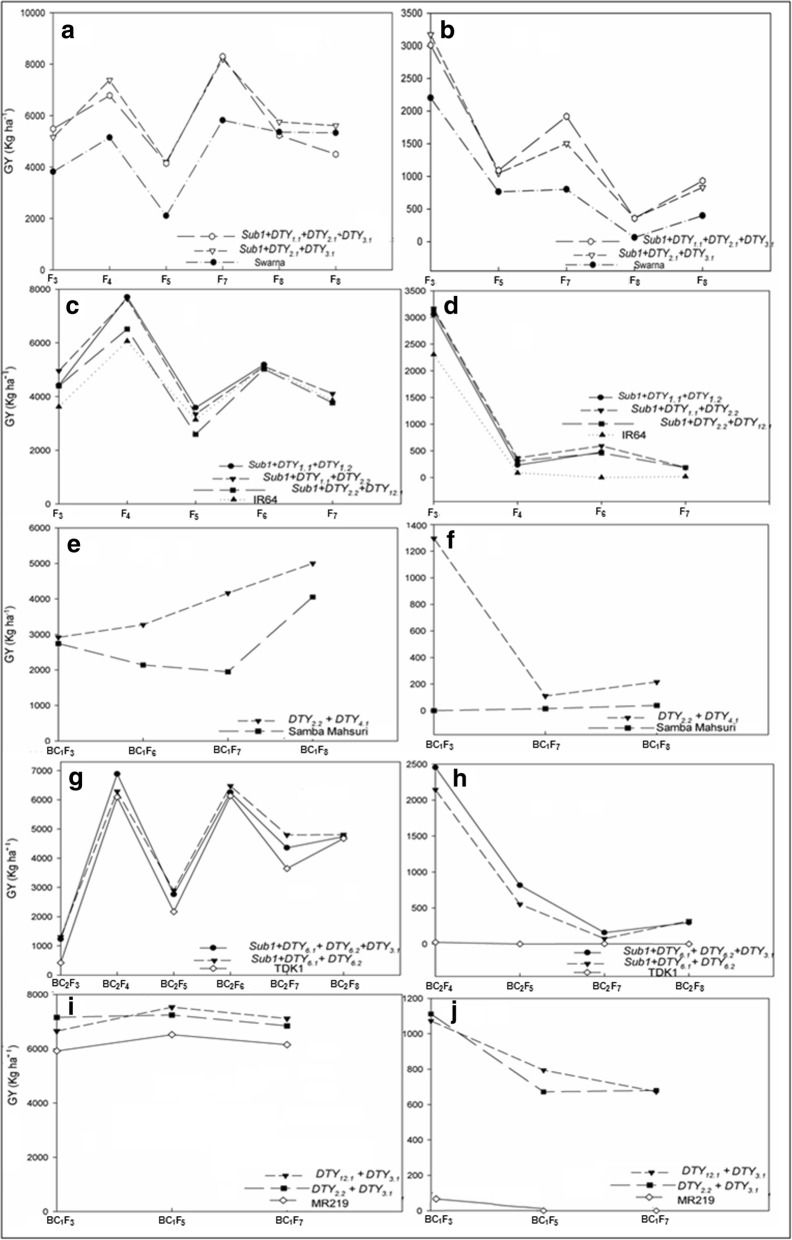
**a** Graph representing the generation (X axis) and mean grain yield (Y axis) of selected SwarnaSub1 pyramided lines under NS (control); **b** Graph representing the generation (X axis) and mean grain yield (Y axis) of selected SwarnaSub1 pyramided lines under RS drought stress; **c** Graph representing the generation (X axis) and mean grain yield (Y axis) of selected IR64Sub1 pyramided lines under NS (control); **d** Graph representing the generation (X axis) and mean grain yield (Y axis) of selected IR64Sub1 pyramided lines under RS drought stress; **e** Graph representing the generation (X axis) and mean grain yield (Y axis) of selected Samba Mahsuri pyramided lines under NS (control); **f** Graph representing the generation (X axis) and mean grain yield (Y axis) of selected Samba Mahsuri pyramided lines under RS drought stress; **g** Graph representing the generation (X axis) and mean grain yield (Y axis) of selected TDK1Sub1 pyramided lines under NS (control); **h** Graph representing the generation (X axis) and mean grain yield (Y axis) of selected TDK1Sub1 pyramided lines under RS drought stress; **i** Graph representing the generation (X axis) and mean grain yield (Y axis) of selected MR219 pyramided lines under NS (control); and (**j**) Graph representing the generation (X axis) and mean grain yield (Y axis) of selected MR219 pyramided lines under RS drought stress

The pyramided lines with either a single gene or in combination of genetic loci associated with grain yield under drought produced a grain yield advantage over the recipient parent across backgrounds and generations (Fig. 1a to j). The pyramided lines with two or more QTLs had shown a high grain yield advantage in Swarna-Sub1 (Table 1), IR64-Sub1 (Table 2), Samba Mahsuri (Table 3), TDK1-Sub1 (Table 4), and MR219 (Table 5) backgrounds. In a Swarna-Sub1 background, a grain yield advantage of 76.2–2478.5 kg ha^− 1^ and 395.7–2376.3 kg ha^− 1^ under non-stress (NS) in *Sub1* + *qDTY*_*1.1*_ *+ qDTY*_*2.1*_ + *qDTY*_*3.1*_ and *Sub1* + *qDTY*_*2.1*_ + *qDTY*_*3.1*_ pyramided lines, respectively, was observed. Under RS drought stress, a grain yield advantage of 292.4–1117.8 and 284.2–2085.5 kg ha^− 1^ in *Sub1* + *qDTY*_*1.1*_ *+ qDTY*_*2.1*_ + *qDTY*_*3.1*_ and *Sub1* + *qDTY*_*2.1*_ + *qDTY*_*3.1*_ pyramided lines, respectively, was observed (Table 1). In an IR64-Sub1 background, the pyramided lines (*Sub1* + *qDTY*_*1.1*_ + *qDTY*_*2.2*_) showed a grain yield advantage ranging from 21.3 to 1571.4 kg ha^− 1^ and 170.4 to 864.7 kg ha^− 1^ under NS and RS drought stress, respectively. Under RS drought stress, the pyramided lines (*Sub1* + *qDTY*_*3.2*_ + *qDTY*_*2.3*_ + *qDTY*_*12.1*_) showed a grain yield advantage of 217.1 to 719.1 kg ha^− 1^ in an IR64-Sub1 background (Table 2). The grain yield advantage ranged from 48.0 to 2216.9 kg ha^− 1^ and 95.5 to 1296.4 kg ha^− 1^ under NS and RS drought stress conditions, respectively, in Samba Mahsuri introgressed with *qDTY*_*2.2*_ *+ qDTY*_*4.1*_ (Table 3). In TDK1-Sub1 pyramided lines (*Sub1 + qDTY*_*3.1*_ + *qDTY*_*6.1*_ + *qDTY*_*6.2*_), the grain yield advantage ranged from 65.2 to 792.0 kg ha^− 1^ and 155.9 to 2429.5 kg ha^− 1^ under NS and RS drought stress conditions, respectively (Table 4). The pyramided lines with *qDTY*_*12.1*_ + *qDTY*_*3.1*_ and *qDTY*_*2.2*_ + *qDTY*_*3.1*_ showed a grain yield advantage of 735.1–1012.8 kg ha^− 1^ and 324.0–1240.9 kg ha^− 1^, respectively, under NS and 672.3–1059.5 kg ha^− 1^ and 571.4–1099.3 kg ha^− 1^, respectively, under RS drought stress conditions in an MR219 background (Table 5).

**Table 1:** Mean comparison of QTL classes of grain yield (kg ha^− 1^) across F_3_ to F_8_ generations under reproductive-stage drought stress and irrigated non-stress control conditions in Swarna-Sub1 background at IRRI, Philippines

QTL class	QTL	2012DS	2012DS	2012DS	2012DS	2012DS	2012WS	2013DS	2013DS	2014DS	2014DS	2015WS	2015WS	2016DS
		NS_Med	RS_Med	RS_ Med	NS_Late	RS_Late	NS	NS	RS	NS	RS	NS	RS	RS
		F_3_	F_3_	F_3_	F_3_	F_3_	F_4_	F_5_	F_5_	F_7_	F_7_	F_8_	F_8_	F_8_
Population size														
		663	366	304	91	84	754	432	432	432	432	52	52	52
A	*qDTY*_*1.1*_	4906 bc	2677 cde	2894 bcf	6766 gh	3674 c	3925 bc	–	–	–	–	–	–	–
B	*Sub1+ qDTY*_*1.1*_	5431 efg	2228ab	2930 bg	4141 a	3652 bc	3536 bcd	–	–	–	–	5191 c	68.24 a	579 b
C	*DTY*_*2.1*_	4811cde	2828 efg	2962 abg	4265 ab	3719 bc	4176 abc	–	–	–	–	–	–	–
D	*Sub1+ qDTY*_*2.1*_	5084 cf	2452 bcde	2776 abde	4649 ab	3554 bc	2729 a	4109 bc	793 ac	–	–	–	–	–
E	*qDTY*_*3.1*_	5098 cdeg	3010 gh	3001 bg	4987 ac	2658 b	–	4135 bc	973 ac	7941 ab	1868 cd	–	–	–
F	*Sub1+ qDTY*_*3.1*_	4705 bc	3027 fh	2984 bg	–	3315 bc	4663 ac	4107 cd	1097 cd	7934 b	1838 cd	4940 b	97.96 a	677 c
G	*Sub1*	5430 cf	2642 bcefh	2334 ab	5338 bcd	3204 bc	3515 a	2948 abc	530 ac	–	–	–	–	–
H	*qDTY*_*1.1*_ *+ qDTY*_*2.1*_	5394 df	2653 ce	3131 efg	6445 fg	3671 c	4308 ab	–	–	–	–	–	–	–
I	*Sub1 + qDTY*_*1.1*_ *+ qDTY*_*2.1*_	5444 ef	2428 ac	3133 efg	6642 fgh	3636 c	4460 ab	3710 bc	605 ab	–	–	–	–	–
J	*qDTY*_*1.1*_ *+ qDTY*_*3.1*_	4788 c	2693 de	2945 be	6395 fg	3481 bc	4288 ab	–	–	–	–	–	–	–
K	*Sub1 + qDTY*_*1.1*_ *+ qDTY*_*3.1*_	4989 cd	2832 efg	3003 ceg	6639 efh	3377 bc	5183 c	3456 b	677 ad	–	–	4676 a	159.19 b	566 b
L	*qDTY*_*2.1*_ *+ qDTY*_*3.1*_	5265 bdf	2998 fh	2955 bg	–	3620 bc	4623 ac	4116 cd	992 bcd	7932 ab	1672 bc	–	–	–
M	*qDTY*_*2.1*_ *+ qDTY*_*3.1*_ *+ Sub1*	5154 cf	3172 h	3162 efg	7380 hi	3714 bc	–	4192 cd	1048 bcd	8194 b	1503 ab	5754 g	360.16 c	830 d
N	*qDTY*_*1.1*_ *+ qDTY*_*2.1*_ *+ qDTY*_*3.1*_	5055 cd	2845 df	3130 dg	7373 hi	3505 c	4807 bc	3912 bd	1073 c	8043 b	1854 d	–	–	–
O	*Sub1+ qDTY*_*1.1*_ *+ qDTY*_*2.1*_ *+ qDTY*_*3.1*_	5484 ef	3010 gh	3167 fg	6780 gh	3859 c	4838 bc	4141 c	1092 c	8297 b	1918 d	5434 e	356.81 c	931 d
X	Parent	3818 a	2203 ab	2465 a	5827 cde	2828 ab	5146 c	2106 a	764 ac	5818 a	799 a	5358 f	64.45 a	398 a
Trial mean		5077	2691	2937	6044	3474	4760	3615	838	7878	1652	5222	175	605
	F- value	3.68	7.39	2.45	19.77	1.21	6.04	13.22	1.79	6.88	3.75	5.38	6.16	3.93
	*p*-value	0.0168	<.0001	0.0018	0.0001	0.2838	<.0001	0.0003	0.0559	0.0003	0.0008	<.0001	0.2991	0.368

The letter display are QTL class labels ordered by mean grain yield of QTL class. Means followed by the same letter (within a column) are not significantly different, *DS* dry season, *WS* wet season, *NS* non-stress, *RS* reproductive-stage drought stress, Med medium duration, Late late duration, X recipient parent (no QTL)

**Table 2:** Mean comparison of QTL classes of grain yield (kg ha^− 1^) across F_3_ to F_7_ generations under reproductive-stage drought stress and irrigated non-stress control conditions in IR64-Sub1 background at IRRI, Philippines

QTL class	QTL	2013WS	2013WS	2014DS	2014DS	2014WS	2015DS	2015DS	2015WS	2015WS
		NS	RS	NS	RS	NS	NS	RS	NS	RS
		F_3_	F_3_	F_4_	F_4_	F_5_	F_6_	F_6_	F_7_	F_7_
	Population size									
		467	467	194	194	64	64	64	18	18
A	*Sub1+ qDTY*_*1.1*_ *+ qDTY*_*1.2*_ *+ qDTY*_*12.1*_	4137 ac	3621 cde	7553 bdf	584 g	–	–	–	–	–
B	*Sub1+ qDTY*_*1.1*_ *+ qDTY*_*1.2*_ *+ qDTY*_*2.2*_ *+ qDTY*_*12.1*_	3640 ac	2605 a	7968 bdf	196 abc	–	–	–	–	–
C	*Sub1+ qDTY*_*1.1*_ *+ qDTY*_*1.2*_ *+ qDTY*_*2.2*_	4986 c	2734 ab	5996 abc	377def	–	–	–	–	–
D	*Sub1+ qDTY*_*1.1*_ *+ qDTY*_*1.2*_	4418 cd	3054 abc	7709 cef	232 abc	3585 ab	5192 a	477 bcd	–	–
E	*Sub1 + qDTY*_*1.1*_ *+ qDTY*_*2.2*_ *+ qDTY*_*12.1*_	3589 ac	2634 abc	–	273 be		3976 a	420 bce	–	–
F	*Sub1 + qDTY*_*1.1*_ *+ qDTY*_*2.2*_	4953 ac	3169 abe	7637 bdf	367 ceg	3347 ab	5120 a	592 bf	4105 a	188 a
G	*Sub1 + qDTY*_*1.1*_	4413 ac	2677 ab	8224 cef	410 eg	–	–	–	–	–
H	*Sub1 + qDTY*_*1.2*_ *+ qDTY*_*12.1*_	4001 ac	2963 abc	6660 abe	245 be	–	5468 a	252 ab	–	–
I	*Sub1 + qDTY*_*1.2*_*+ qDTY*_*2.2*_ *+ qDTY*_*12.1*_	5370 cb	3352 abe	8790 bf	259 be	–	–	–	–	–
J	*Sub1 + qDTY*_*12.1*_	4380 cd	2690 abd	6117 ab	189 bc	3066 ab	5125 a	372 abc	3997 a	64 a
K	*Sub1 + qDTY*_*2.2*_ *+ qDTY*_*12.1*_	4395 cd	3130 bc	6512 ab	308 ae	2592 a	5026 a	459 bc	3762 a	186 a
L	*Sub1 + qDTY*_*2.2*_	4252 cd	3767 e	7893 cf	223 abc	–	–	–	–	–
M	*Sub1 + qDTY*_*2.3*_ *+ qDTY*_*12.1*_	3168 ac	3084 abe	8532 cef	194 be	–	–	–	–	–
N	*Sub1 + qDTY*_*2.3*_	3145 ab	2602 a	7080 bde	244 abcd	–	–	–	–	–
O	*Sub1 + qDTY*_*3.2*_ *+ qDTY*_*12.1*_	3670 ac	2746 abd	7145 abf	263 bef	–	–	–	–	–
P	*Sub1 + qDTY*_*3.2*_ *+ qDTY*_*2.2*_ *+ qDTY*_*12.1*_	3109 ac	2728 abd	7798 bdf	197 be	–	–	–	–	–
Q	*Sub1 + qDTY*_*3.2*_ *+ qDTY*_*2.2*_	3055abd	2526 a	6441 ab	220 abcd	2381 a	4398 a	761 f	–	–
R	*Sub1 + qDTY*_*3.2*_ *+ qDTY*_*2.3*_ *+ qDTY*_*12.1*_	2845 ac	2931 abc	6469 abc	304 abcd	2293 a	4570 a	719 def	3883 a	275 a
S	*Sub1 + qDTY*_*3.2*_ *+ qDTY*_*2.3*_	1688 a	2891 abe	5319 a	304 bef	–	4727 a	255 ab	–	–
T	*Sub1 + qDTY*_*3.2*_	3444 ac	3427 be	6230 ad	124 b	–	–	–	–	–
X	Parent	3620 ac	2305 a	6066 abf	87 abc	3139 ab	5099 a	0a	3849 a	18 a
Trial mean		3853	2998	7181	277	3024	4870	862	3943	128
	F- value	1.59	2.88	2.92	3.22	2.83	2.26	4.32	1.54	1.53
	p-value	0.2956	0.006	0.0006	0.0011	0.0363	0.404	0.0004	0.5566	0.5585

The letter display are QTL class labels ordered by mean grain yield of QTL class. Means followed by the same letter (within a column) are not significantly different, *DS* dry season, *WS* wet season, *NS* non-stress, *RS* reproductive-stage drought stress, X recipient parent (no QTL)

**Table 3:** Mean comparison of QTL classes of grain yield (kg ha^−1^) across BC_1_F_3_ to BC_1_F_8_ generations under reproductive-stage drought stress and irrigated non-stress control conditions in Samba Mahsuri background at IRRI, Philippines

QTL class	QTL	2013DS	2013DS	2014WS	2015WS	2015WS	2016DS	2016DS
		NS	RS	NS	NS	RS	NS	RS
		BC_1_F_3_	BC_1_F_3_	BC_1_F_6_	BC_1_F_7_	BC_1_F_7_	BC_1_F_8_	BC_1_F_8_
Population size								
		42	42	70	20	20	20	20
A	*qDTY*_*2.2*_	2020 a	1069 bc	3405 b	3327 b	44 a	–	–
B	*qDTY*_*4.1*_	1900 a	894 b	3340 b^†^	4727 d^†^	184 b^†^	5643 b^†^	33 a
C	*qDTY*_*2.2*_ *+ qDTY*_*4.1*_	2916 b	1296 c	3270 b	4161 c	110 ba	4999 a	216 b
X	Parent	2742 b	0 a	2137 a	1945 a	15 a	4051 a	39 a
	Trial Mean	2395	815	3038	3540	88	5198	96
F- value		31.22	46.37	11.18	43.03	2.12	19.98	62.66
p-value		0.0089	< 0.0001	< 0.0001	< 0.0001	0.09	< 0.0001	< 0.0001

The letter display are QTL class labels ordered by mean grain yield of QTL class. Means followed by the same letter (within a column) are not significantly different, *DS* dry season, *WS* wet season, *NS* non-stress, *RS* reproductive-stage drought stress, X recipient parent (no QTL), ^†^Mean data of only 2 lines

**Table 4:** Mean comparison of QTL classes of grain yield (kg ha^−1^) across BC_2_F_3_ to BC_2_F_8_ generations under reproductive-stage drought stress and irrigated non-stress control conditions in TDK-Sub1 background at IRRI, Philippines

QTLclass	QTL	2013WS	2014DS		2014WS	2015DS		2015WS		2016DS	
		RS	NS	RS	NS	NS	RS	NS	RS	NS	RS
		BC_2_F_3_	BC_2_F_4_	BC_2_F_4_	BC_2_F_5_	BC_2_F_6_	BC_2_F_6_	BC_2_F_7_	BC_2_F_7_	BC_2_F_8_	BC_2_F_8_
Population size											
		843	231	231	48	48	48	60	60	60	60
A	*Sub1 + qDTY*_*6.1*_ *+ qDTY*_*6.2 +*_ *qDTY*_*3.1*_	1232 gh	6883 bc	2453 c	2763 bc	6252bc	816 f	4356 ab	158 de	4739 ab	298 cd
B	*qDTY*_*6.1*_ *+ qDTY*_*6.2 +*_ *qDTY*_*3.1*_	1298 gh	6289 b	2069 b	2629 ac	6174 c	250 bc	4966 cd	122 cd	4871 ab	278 c
C	*Sub1+ qDTY*_*6.1*_ *+ qDTY*_*6.2*_	1301 gi	6289 abc	2143 bc	2897 bcd	6475 c	552 de	4797 bd	73.83 abc	4804 b	320 cd
D	*Sub1+ qDTY*_*6.1*_ *+ qDTY*_*3.1*_	1091 fde	5707 ab	2120 bc	3476 c	5958 ab	368 bd	4657 bc	75 bc	4780 ab	179 ac
E	*Sub1+ qDTY*_*6.2*_ *+ qDTY*_*3.1*_	1178 ge	6061 abc	2112 bc	2576 ac	5157 a	274 bc	–	–	–	–
F	*qDTY*_*6.1*_ *+ qDTY*_*6.2*_	998 cd	3890 a	2126 bc	2307 ac	4799 a	501 cde	–	–	–	–
G	*qDTY*_*6.1*_ *+ qDTY*_*3.1*_	1012 ge	5874 ab	1959 b	2704 ac	6775 c	211.97 b	5074 d	73 b	4793 ab	113 ab
H	*qDTY*_*6.2*_ *+ qDTY*_*3.1*_	1134 fe	–	–	–	–	–	–	–	–	–
I	*Sub1 + qDTY*_*6.2*_	1051 ce	–	–	–	–	–	–	–	–	–
J	*Sub1+ qDTY*_*6.1*_	1446 j	–	–	–	–	–	–	–	–	–
K	*Sub1 + qDTY*_*3.1*_	1376 hij	–	–	–	–	–	–	–	–	–
L	*qDTY*_*6.2*_	1416 ij	–	–	–	–	–	–	–	–	–
M	*qDTY*_*6.1*_	1308 gh	–	–	–	–	–	–	–	–	–
N	*qDTY*_*3.1*_	1217 fg	–	–	–	–	–	–	–	–	–
X	Parent	421 a	6091 abc	24 a	2167 a	6135 bc	0 a	3647 a	2 a	4674 a	0 a
Trial mean		1165	5886	1863	2715	6091	409	4583	84	4760	198
	F- value	34.1	6.6	1.03	3.21	4.99	16.32	6.44	6.0	5.32	5.0
	p-value	<.0001	0.0012	0.4207	0.0341	0.0105	<.0001	<.0001	0.0001	0.0013	0.0046

The letter display are QTL class labels ordered by mean grain yield of QTL class. Means followed by the same letter (within a column) are not significantly different, *DS* dry season, *WS* wet season, *NS* non-stress, *RS* reproductive-stage drought stress, X recipient parent (no QTL)

**Table 5:** Mean comparison of QTL classes of grain yield (kg ha^−1^) across BC_1_F_3_ to BC_1_F_7_ generations under reproductive-stage drought stress and irrigated non-stress control conditions in MR219 background at IRRI, Philippines

QTL class	QTL	2013DS		2014DS		2015DS	
		NS	RS	NS	RS	NS	RS
		BC_1_F_3_	BC_1_F_3_	BC_1_F_5_		BC_1_F_7_	BC_1_F_7_
Population size							
		214	214	620	620	70	70
A	*qDTY*_*12.1*_	6229 a	654 b	6967 b	301 a	*–*	–
B	*qDTY*_*12.1*_ *+ qDTY*_*2.2*_	6633 b	761 bc	7364 ac	598 b	5986 a	540 c
C	*qDTY*_*12.1*_ *+ qDTY*_*3.1*_	6652 ac	1072 d	7532 cd	794 e	7111 c	672 d
D	*qDTY*_*2.2*_	6760 ab	904 cd	7079 ba	669 bc	6957 c	393 b
E	*qDTY*_*2.2*_ *+ qDTY*_*3.1*_	7158 bc	1112 d	7243 cd	663 c	6843 bc	679 d
F	*qDTY*_*2.2*_ *+ qDTY*_*3.1*_ *+ qDTY*_*12.1*_	6799 ab	642 b	7106 ad	442 b	6674 bc	578 cd
G	*qDTY*_*3.1*_	6488 a	890 c	7374 ac	568 c	6923 bc	537 bcd
X	Parent	5917 ab	13 a	6519 b	0 ab	6148 ab	0 a
Trial mean		6705	781	7173	505	6663	486
F- value		2.0	11.76	9.45	19.39	7.76	6.18
p-value		0.05	< 0.0001	< 0.0001	< 0.0001	0.0004	<.0001

The letter display are QTL class labels ordered by mean grain yield of QTL class. Means followed by the same letter (within a column) are not significantly different, *DS* dry season, *WS* wet season, *NS* non-stress, *RS* reproductive-stage drought stress, X recipient parent (no QTL)

### Performance of pyramided lines in the F_3_ generation

Mean performances of QTL classes from F_3_ to F_7_/F_8_ of Swarna-Sub1, IR64-Sub1, Samba Mahsuri, TDK1-Sub1, and MR219 pyramided lines are shown in Tables 1, 2, 3, 4, and 5, respectively.

In a Swarna background, two classes (*Sub1 + qDTY*_*1.1*_ *+ qDTY*_*2.1*_ + *qDTY*_*3.1*_ and *Sub1 + qDTY*_*2.1*_ + *qDTY*_*3.1*_) showed higher performance in F_3_ under both NS and RS drought stress (Table 1). In an IR64-Sub1 background, three classes (*Sub1* + *qDTY*_*1.1*_ + *qDTY*_*1.2*_, *Sub1* + *qDTY*_*1.1*_ + *qDTY*_*2.2*_, *Sub1 + qDTY*_*2.2*_ + *qDTY*_*12.1*_) showed higher performance under NS and RS drought stress both, whereas *Sub1* + *qDTY*_*3.2*_ + *qDTY*_*2.3*_ + *qDTY*_*12.1*_ performed better under RS drought stress only in F_3_ (Table 2). In Samba Mahsuri background, the QTL class *qDTY*_*2.2*_ + *qDTY*_*4.1*_ showed a higher performance than a single QTL under both NS and RS drought stress in F_3_ (Table 3). In a TDK1-Sub1 background, the classes consisting of pyramided lines with *Sub1* + *qDTY*_*3.1*_ + *qDTY*_*6.1*_ + *qDTY*_*6.2*_ and *Sub1* + *qDTY*_*6.1*_ + *qDTY*_*6.2*_ showed a stable and high effect across variable growing conditions in F_3_ (Table 4). In the MR219 background, pyramided lines having *qDTY*_*12.1*_ + *qDTY*_*3.1*_ and *qDTY*_*2.2*_ + *qDTY*_*3.1*_ showed significant yield advantage under both NS and RS drought stress (Table 5).

### Validation of MAB-selected class performance in subsequent generations

The performance of pyramided line classes identified as superior in the F_3_ generation was found to be consistent and higher than other QTL classes throughout F_4_, F_5_, F_6_, F_7,_ and F_8_ generations (except where the number of lines per class was less) across all five studied backgrounds in the present study. The high mean grain yield QTL classes in the F_3_ generation, *Sub1* + *qDTY*_*1.1*_ *+ qDTY*_*2.1*_ + *qDTY*_*3.1*_ and *Sub1* + *qDTY*_*2.1*_ + *qDTY*_*3.1*_ in a Swarna background (Table 1), *qDTY*_*2.2*_ + *qDTY*_*4.1*_ in a Samba Mahsuri background (Table 3), and *Sub1 + qDTY*_*3.1*_ + *qDTY*_*6.1*_ + *qDTY*_*6.2*_ and *Sub1 + qDTY*_*6.1*_ + *qDTY*_*6.2*_ in a TDK1-Sub1 background (Table 4) had maintained their high mean grain yield performance from the F_4_ to F_8_ generations over other QTL classes. The low mean yield performers in the F_3_ generation, *Sub1 + qDTY*_*1.1*_*, Sub1 + qDTY*_*1.1*_ *+ qDTY*_*3.1*_ in a Swarna-Sub1 background (Table 1)*, qDTY*_*2.2*_ in a Samba Mahsuri background (Table 3), and *qDTY*_*6.1*_ *+ qDTY*_*3.1*_ and *Sub1 + qDTY*_*6.2*_ *+ DTY*_*3.1*_ in a TDK1-Sub1 background (Table 4), were observed to be lower yielders in each of the generations from F_4_ to F_8_. The significant high grain yield advantage of *Sub1 + qDTY*_*1.1*_ + *qDTY*_*1.2*_, *Sub1 + qDTY*_*1.1*_ + *qDTY*_*2.2*_, *Sub1 + qDTY*_*2.2*_ + *qDTY*_*12.1*_, and *Sub1 + qDTY*_*3.2*_ + *qDTY*_*2.3*_ + *qDTY*_*12.1*_ in an IR64-Sub1 background (Table 2) and of *qDTY*_*12.1*_ + *qDTY*_*3.1*_ and *qDTY*_*2.2*_ + *qDTY*_*3.1*_ in an MR219 background (Table 5) was consistent from the F_4_ to F_7_ generation. QTL classes *Sub1 + qDTY*_*1.2*_ + *qDTY*_*12.1*_, *Sub1* + *qDTY*_*3.2*_ + *qDTY*_*2.3*_, and *qDTY*_*1.1*_ *+ qDTY*_*2.2*_ *+ qDTY*_*12.1*_ *+ Sub1* in an IR64-Sub1 background showed lower yield from F_3_ to subsequent generations (Table 2). The low grain yield performance of *qDTY*_*12.1*_ *+ qDTY*_*2.2*_ and *qDTY*_*2.2*_ *+ qDTY*_*3.1*_ *+ qDTY*_*12.1*_ under RS drought stress in MR219 was maintained from the F_4_ to F_7_ generation (Table 5). None of the inferior QTL classes identified in F_3_ outperformed the identified superior QTL combination class or combination classes in any advanced generation under NS as well as under variable intensities of RS drought stress in different seasons/years across generations from F_4_ to F_7_/F_8_.

### Cost effectiveness of the early generation selection

The genotyping cost for the whole population considering all QTL classes from F_3_ to F_7_/F_8_ ranged from USD 9225 to USD 21760 whereas the genotyping cost accounting for further advancement and screening (F_4_ to F_7_/F_8_) of only superior classes in F_3_ varied from USD 5730 to USD 8978 (Table 6). A genotyping cost savings of USD 12443, 3720, 14,780, 2273, and 6225 was observed in Swarna-Sub1, IR64-Sub1, Samba Mahsuri, TDK1-Sub1, and MR219 backgrounds, respectively, with a range of savings of USD 2273 to USD 14780 in all five backgrounds.

**Table 6:** Comparison of genotyping cost (USD) considering advancement of all QTL classes versus advancement of only higher performing F_3_ generation QTL classes

Background	Generation	Number of QTL classes	Population size		Cost (USD)		Total genotyping cost (USD)		Savings (USD)
			Based on all classes	Based on selected classes	Based on all classes	Based on selected classes	Based on all classes	Based on selected classes	
Swarna-Sub1	F_3_	15	754	754	5655	5655	21,420	8978	12,443
	F_4_	15	754	106	5655	795			
	F_5_	10	432	106	3240	795			
	F_6_	10	432	106	3240	795			
	F_7_	6	432	108	3240	810			
	F_8_	5	52	17	390	127.50			
IR64-Sub1	F_3_	20	467	467	7005	7005	12,105	8385	3720
	F_4_	19	194	46	2910	690			
	F_5_	19	64	18	960	270			
	F_6_	13	64	18	960	270			
	F_7_	7	18	10	270	150			
Samba Mahsuri	BC_1_F_3_	3	42	42	210	210	21,760	6980	14,780
	BC_1_F_4_	3	3000	640	15,000	3200			
	BC_1_F_5_	3	1200	640	6000	3200			
	BC_1_F_6_	3	70	44	350	220			
	BC_1_F_7_	2	20	15	100	75			
	BC_1_F_8_	2	20	15	100	75			
TDK1-Sub1	BC_2_F_3_	14	843	843	6323	6323	9225	6954	2272
	BC_2_F_4_	7	231	43	1733	323			
	BC_2_F_5_	7	48	14	360	105			
	BC_2_F_6_	7	48	14	360	105			
	BC_2_F_7_	5	60	13	450	98			
MR219	BC_1_F_3_	7	214	214	1605	1605	11,955	5730	6225
	BC_1_F_4_	7	620	240	4650	1800			
	BC_1_F_5_	7	620	240	4650	1800			
	BC_1_F_6_	7	70	35	525	262.50			
	BC_1_F_7_	7	70	35	525	262.50			

The genotyping cost was calculated considering five markers per QTL (one peak/near the peak, two right-hand-side flanking markers, and two left-hand-side flanking markers) and USD 0.50 per data point

The phenotyping cost for the whole population ranged from USD 29197 to USD 157455 whereas it was USD 20225 to USD 50507 in the case of selected classes (Table 7). A phenotyping cost savings of USD 60023, 8973, 10,963, 106,948, and 30,029 was observed in Swarna-Sub1, IR64-Sub1, Samba Mahsuri, TDK1-Sub1, and MR219 backgrounds, respectively, with phenotyping cost savings of USD 8973–106,948 in all five backgrounds. The genotyping and phenotyping cost and savings were high in Samba Mahsuri as the number of plant samples in the whole population set in the F_4_ generation was more than in the QTL class selected in F_3_ (*DTY*_*2.2*_ *+ DTY*_*4.1*_) (Table 6). The cost savings was inversely proportional to the number of QTL combination classes identified as providing superior performance in F_3_.

**Table 7:** Comparison of phenotyping cost (USD) considering advancement of all QTL classes versus advancement of only higher performing F_3_ generation QTL classes

Background	Generation	Population size		Phenotyping cost (USD)		Total phenotyping cost (USD)		Savings (USD)
		Based on all classes	Based on selected classes	Based on all classes	Based on selected classes	Based on all classes	Based on selected classes	
Swarna-Sub1	F_3_	754	754	27,280	27,280	103,330	43,307	60,023
	F_4_	754	106	27,280	3835			
	F_5_	432	106	15,630	3835			
	F_6_	432	106	15,630	3835			
	F_7_	432	108	15,630	3907			
	F_8_	52	17	1881	615			
IR64-Sub1	F_3_	467	467	16,896	16,896	29,197	20,225	8973
	F_4_	194	46	7019	1664			
	F_5_	64	18	2316	651			
	F_6_	64	18	2316	651			
	F_7_	18	10	651	362			
Samba Mahsuri	BC_1_F_3_	42	42	1520	1520	157,455	50,507	106,948
	BC_1_F_4_	3000	640	108,540	23,155			
	BC_1_F_5_	1200	640	43,416	23,155			
	BC_1_F_6_	70	44	2533	1592			
	BC_1_F_7_	20	15	724	543			
	BC_1_F_8_	20	15	724	543			
TDK1-Sub1	BC_2_F_3_	843	843	30,500	30,500	44,501	33,539	10,963
	BC_2_F_4_	231	43	8358	1556			
	BC_2_F_5_	48	14	1737	507			
	BC_2_F_6_	48	14	1737	507			
	BC_2_F_7_	60	13	2171	470			
MR219	BC_1_F_3_	214	214	7743	7743	57,671	27,642	30,029
	BC_1_F_4_	620	240	22,432	8683			
	BC_1_F_5_	620	240	22,432	8683			
	BC_1_F_6_	70	35	2533	1266			
	BC_1_F_7_	70	35	2533	1266			

The phenotyping cost of USD 36.18 per entry was calculated considering two replications and screening under NS and RS drought stress with plot size of 1.54 m^2^ (IRRI Standard drought screening costing)

### Interaction among QTLs and with background

In our study, *qDTY*_*1.1*_ showed positive interactions with *qDTY*_*2.1*_*, qDTY*_*2.2*_*,* and *qDTY*_*3.1*_*,* whereas *qDTY*_*2.2*_ showed positive interactions with *qDTY*_*4.1*_*, qDTY*_*12.1*_*,* and *qDTY*_*3.1*_*. qDTY*_*3.1*_ showed positive interactions with *qDTY*_*1.1*_*, qDTY*_*2.2*_*, qDTY*_*12.1*_*, qDTY*_*6.1*_*,* and *qDTY*_*6.2*_ at least in the genetic backgrounds that we studied in the present experiment. Such information will be helpful to breeders in selecting QTL combinations in their MAB programs.

## Discussion

### Phenotypic evaluation of QTLs pyramided lines

**Fig. 2:**
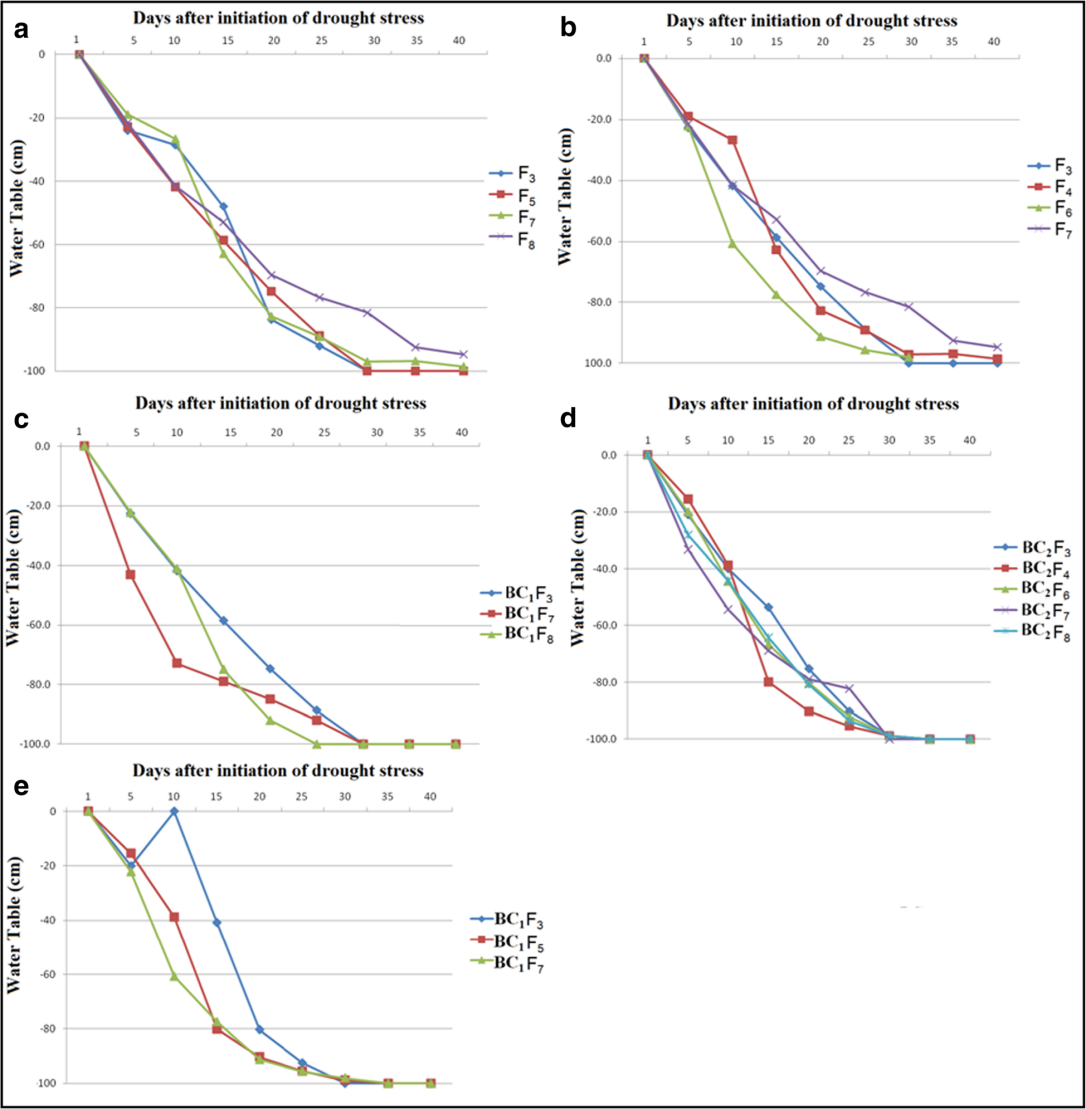
Soil water potential measured by parching water table level in experiments (**a**) Swarna-Sub1 pyramided lines with *qDTY*_*1.1*_*, qDTY*_*2.1*_, and *qDTY*_*3.1*_ in different generations; **b** IR64-Sub1 pyramided lines with *qDTY*_*1.1*_*, qDTY*_*1.2*_*, qDTY*_*2.2*_*, qDTY*_*2.3*_*, qDTY*_*3.2*_, and *qDTY*_*12.1*_ in different generations; **c** Samba Mahsuri pyramided lines with *qDTY*_*2.2*_ and *qDTY*_*4.1*_ in different generations; **d** TDK1-Sub1 pyramided lines with *qDTY*_*3.1,*_ *qDTY*_*6.1,*_ and *qDTY*_*6.2*_ in different generations; and (**e**) MR219 pyramided lines with *qDTY*_*2.2*_*, qDTY*_*3.1,*_ and *qDTY*_*12.1*_ in different generations using polyvinyl chloride (PVC) pipe

The yield reduction in RS drought stress experiments was 45, 77, 79, and 97% in F_3_, F_5_, F_7_, and F_7_ generations, respectively, in Swarna-Sub1 introgression lines as compared to the mean yield of the NS experiments. In IR64-Sub1, the yield reduction was 22, 96, 82, and 97% in F_3_, F_4_, F_6_, and F_7_ generations, respectively. In the Samba Mahsuri background, the mean yield reduction was 66, 98, and 98% in F_3_, F_7_, and F_8_ generations, respectively, in the RS drought stress experiment compared with NS experiments. A grain yield reduction of 68, 93, 98, and 96% was observed in F_4_, F_6_, F_7_, and F_8_ generations, respectively, under RS drought stress compared with NS in TDK1-Sub1 introgressed lines. In MR219 introgressed lines, the yield reduction under RS drought stress compared with NS was 88, 93, and 93% in F_3_, F_5_, and F_7_ generations, respectively. Accurate standardized phenotyping under RS drought stress assists breeders in rejecting inferior QTL classes in F_3_ itself and is the basis of success of the combined MAS breeding approach. It is evident from the yield reduction as well as the water table depths (Fig. 2a-e) that the stress level in RS drought stress experiments ranged from moderate to severe drought stress intensity at the reproductive stage in most of the cases. DTF of majority of pyramided lines was less than that of recipient lines under RS but not under NS. Some of the selected progenies showed early DTF than recipient under NS and this may have resulted from linkages of the drought QTLs with earliness (Vikram et al. 2016). Most of the progenies showed similar PHT as that of recipient cultivars under NS but higher PHT under RS because of their increased ability to produce biomass under RS (data not presented).

### Selection of superior QTLs class in early generation

In a marker-assisted QTL introgression/pyramiding program, it would be very valuable to explore QTL combinations with high performance in early generations. The F_2_ generation is highly heterogeneous; therefore, screening of a large population size is essential to maximize the exploitation of genetic variation (Kahani and Hittalmani 2015). Sometimes, based on the availability of resources, fields for phenotyping, as well as capacity of breeding programs, breeders have to reduce the population size, which may lead to a loss of existing positive genetic variability in the population (Govindaraj et al. 2015). In the present study, the screening of a large-sized F_3_ population was carried out under control (NS) and RS drought stress conditions. The classification of the population in different classes based on QTL combinations in each generation (F_3_ to F_7_/F_8_) followed by class analysis to see the performance of each QTL class across generation advancement proved to be an effective approach in identifying best-bet QTL combination classes across five high-yielding genetic backgrounds. The performance of the genotypes in a particular QTL class was consistent from F_3_ to F_7_/F_8_ generations in all five studied background in the present study. The advancement of the classes with high mean grain yield performance in the F_3_ generation in addition to the MAB approach involving stepwise phenotyping and genotyping screening suggested this as being a cost/labor- and resource-effective breeding strategy. The lesser number of genotypes in advanced generations can be screened more precisely in a large plot size with more replications. The current cost-effective high-throughput phenotyping platform (Comar et al. 2012; Andrade-Sanchez et al. 2014; Sharma and Ritchie 2015; Bai et al. 2016) can be used for precise breeding and physiological studies considering the small population size. Even at the F_3_ level, some heterozygosity will be observed when more genes are involved in the introgression program. However, in our study, we did not observe any change in performance of QTL classes found superior in F_3_, indicating the F_3_ generation to be suitable to conduct class analysis and reject inferior classes.

### Population size and validation of combined breeding strategy

In addition to the modern next-generation genotyping strategies (Barba et al. 2014; Rius et al. 2015; Dhanapal and Govindaraj 2015) and agricultural system models (Antle et al. 2016), several breeding strategies involving correlated traits as selection criteria in early generations (Senapati et al. 2009), grain yield (Kumar et al. 2014), secondary traits (Mhike et al. 2012), genetic variance, heritability (Almeida et al. 2013), path coefficient analysis, selection tolerance index (Dao et al. 2017), and yield index (Raman et al. 2012) have been suggested for use in breeding programs. The consistent performance of pyramided lines with specific QTL combinations across generations (F_3_ to F_7_/F_8_) in five backgrounds in the present study validates the potential of the suggested combined MAS breeding approach presented in the current study. The integration of accurate phenotyping and the selection of the best class representing the genetic variability of the whole population in early generations are critical steps for the practical implementation of this ultimate novel breeding strategy. Keeping a large F_3_ population size depending upon the number of genes/QTLs being introgressed and precise phenotyping to exploit the hidden potential of each genotype in each QTL class could maximize the potential output of each class in early generations. The most logical QTL-class performance-derived novel breeding strategy could be adopted to optimize the breeding efficiency of small-to moderate-sized breeding programs in rice breeding improvement programs. Further, the strategy could be equally useful to other crops in which major genes/QTLs determine the expression of traits and QTL x QTL or QTL x genetic background interactions have been identified.

We were able to understand the effectiveness of early generation selection in the marker-assisted introgression program for drought because the breeding program maintained systematic data for both genotyping and phenotyping conducted over the past six or more years. It was only after we successfully identified the best lines coming from each introgression program after successful multi-location evaluation that we realized that, as the breeding program will need to bring in more and more genes for multiple traits to address each of the new emerging climate-related challenges, modifications that allow plant breeders to make large-scale rejections in the early generation will become necessary. The effectiveness of the combined MAS strategy is evident from the result that, in none of the five cases were the superior QTL class combinations identified in F_3_ outperformed by inferior classes identified in F_3_ in any advanced generation under both NS and variable intensities of RS drought stress in different seasons/years across generations from F_4_ to F_6_/F_7_/F_8_.

### Cost-effectiveness of combined breeding strategy

Breeding practices are challenged by being laborious, time consuming, and non-economical, requiring large land space and a large population size (Sandhu and Kumar 2017), being imprecise, and having unreliable phenotyping screening (Bhat et al. 2016); hence, an economical, fast, accurate, and efficient breeding selection system is required to increase grain yield potential and productivity (Khan et al. 2015). The cost-benefit balance (Bhat et al. 2016) must be considered in increasing genetic gain in the new era of modern science. The use of the class analysis approach in the F_3_ generation followed by advancing only higher performing classes reported a genotyping cost savings of 25–68% and phenotyping cost savings of 25–68% compared with the traditional molecular marker breeding approach (Table 6). Although the cost-benefit of the combined MAS breeding strategy will always be inversely proportional to the number of superior QTL class combinations identified for advancement in F_3_ and subsequent generations, the cost savings will increase as the number of genes included in the introgression program increases because of the rejection of a larger proportion of the total population early in the F_3_ generation. This procedure will save time, labor, resources, and space and will allow breeders to focus only on germplasm with higher value. This will reduce the population size for phenotypic and genotypic selection in advanced generations compared with earlier marker-assisted breeding strategies (Price 2006; McNally et al. 2009; Yadaw et al. 2013; Sandhu et al. 2014; Brachi et al. 2012; Begum et al. 2015). It will be practical and realistic only if the phenotyping, genotyping, and class analysis in early generations are accurate.

### Interactions among QTLs and with background

The QTLs for grain yield under drought have shown QTL x QTL (Sandhu et al. 2018) as well as QTL x genetic background interactions (Dixit et al. 2012a, b; Sandhu et al. 2018). Many such interactions that may occur between QTL x QTL and QTL x genetic background are unknown. Such positive/negative interactions affecting grain yield under normal or RS situation can be captured through approach that combines selection based on phenotyping and genotyping in the early generations. The current study clearly demonstrated the success of selection based on combining phenotyping and genotyping in identifying better progenies in early generation thereby reducing the number of progenies to be advanced. Number of plants to be generated and evaluated in the early generations will depend upon the number of QTLs/genes to be introgressed together, size of introgressed QTLs region as well as availability of closely linked markers for each of the QTLs. The QTLs for grain yield under drought have shown undesirable linkages with low yield potential, very early maturity duration, tall plant height (Vikram et al. 2015). At IRRI, studies were undertaken to break the undesirable linkages of QTLs with tall plant height, very early maturity duration and low yield potential (Vikram et al. 2015). Such improved lines were used in the MAS introgression program. The drought tolerant donors N22, Dular, Apo, Way Rarem, Kali Aus, Aday Sel that are source of identified QTLs do not possess good grain quality. Even though, we did not study the linkage of *qDTYs* with grain quality, the introgressed lines released as varieties in IR64, Swarna backgrounds in India and Nepal did not reveal any adverse effect on grain quality. The yield superiority of lines with two or more QTLs under both NS and RS drought stress over the five high-yielding backgrounds clearly indicated that *qDTY* QTLs identified at IRRI are free from undesirable linkage drag and can be successfully used in MAB programs targeting yield improvement under RS drought stress. Further, in Swarna-Sub1, IR64-Sub1, and TDK-Sub1, the highest yielding classes identified were the classes possessing both *Sub1* and combinations of the drought QTLs. The yield superiority of such classes across these three backgrounds over all the generations clearly indicated that tolerance of submergence and drought can be effectively combined even though they are governed by two different physiological mechanisms. In the QTL study undertaken at IRRI, *qDTY*_*1.1*_ showed a significant mean yield advantage in MTU1010 and IR64 (Sandhu et al. 2015); *qDTY*_*2.2*_ in Pusa Basmati 1460, MTU1010, and IR64 (Venuprasad et al. 2007; Swamy et al. 2013; Sandhu et al. 2013; Sandhu et al. 2014); *qDTY*_*2.3*_ in Vandana and IR64 (Dixit et al. 2012b; Sandhu et al. 2014); *qDTY*_*3.2*_ in Sabitri (Yadaw et al. 2013); *qDTY*_*6.1*_ in IR72 (Venuprasad et al. 2009); and *qDTY*_*12.1*_ in Vandana (Bernier et al. 2007), Sabitri (Mishra et al. 2013), Kalinga, and Anjali backgrounds. Similar interaction of *qDTY*_*2.3*_ and *qDTY*_*3.2*_ with *qDTY*_*12.1*_ in a Vandana background (Dixit et al. 2012b); *qDTY*_*2.2*_ and *qDTY*_*3.1*_ with *qDTY*_*12.1*_ in an MRQ74 background (Shamsudin et al. 2016); and *qDTY*_*2.2*_ + *qDTY*_*4.1*_ in an IR64 background (Swamy et al. 2013) was observed. The interaction of identified QTLs with other QTLs in more than two backgrounds supports the usefulness of such QTL classes in MAS. In all five of these cases, through genotyping and phenotyping we were able to identify QTL class combinations with positive interactions and higher yield. As more data are generated across different backgrounds and interactions are established, breeders will have the ability to identify and forward only selected classes without phenotyping from F_3_ onward.

### Pyramiding of multiple QTLs associated with multiple traits

With the identification of gene-based/closely linked markers for different biotic stresses (bacterial blight, blast, brown planthopper, gall midge) and abiotic stresses (submergence, drought, phosphorus deficiency, cold, anaerobic germination, high temperature), the MAB program is moving forward to introgress more genes/QTLs to develop climate-resilient and better rice varieties. For effective tolerance to develop a variety combining tolerance of biotic and abiotic stresses – bacterial leaf blight (three genes – *xa5, xa13, Xa21*), blast (two – *pi2, pi9*), brown planthopper (two – *BPH3, BPH17*), gall midge (two – *Gm4, Gm8*), drought (three –*qDTY*_*1.1*_*, qDTY*_*2.1*_*, qDTY*_*3.1*_), and submergence (*Sub1*) – researchers will need introgression and the combination of 13–15 genes/QTLs in gene combinations mentioned here or in other combinations depending upon the prevalence of a pathotype/biotype in different regions. The number of genes to be introgressed is likely to increase as exposure of rice to high temperature at the reproductive stage will probably increase in most rice-growing regions. The introgression of 10–15 genes will not only require a larger initial population in F_2_ and F_3_ but will also lead to increased positive/negative interactions between genes/QTLs. With capacity development, as more and more breeding programs adopt marker-assisted introgression of more genes, the combined MAS strategy will be of great help to plant breeders in reducing the number of plants that they should handle in each generation and make their breeding program cost-effective.

## Conclusions

The selection of QTL classes with a high mean yield performance and positive interactions among loci and with background in the early generation and consistent performance of QTL classes in subsequent generations across five backgrounds supports the effectiveness of a combined MAS breeding strategy. The challenge ahead is the appropriate estimation of the precise population size to be used for QTL class analysis in the early F_3_ generation to maintain genetic variability as the number of genes/QTLs increases further. Integration of a cost-effective, efficient, designed, statistics-led early generation superior QTL class selection-based breeding strategy with new-era genomics such as genotyping by sequencing and genomic selection could be an important breakthrough to build up a scientific next-generation breeding program.

## Methods

The study was conducted at the International Rice Research Institute (IRRI), Philippines, to introgress QTLs for grain yield under RS drought stress in the background of improved high- yielding widely grown but drought-susceptible varieties from India (Swarna, IR64, Samba Mahsuri), Lao PDR (TDK1), and Malaysia (MR219).

Five sets of introgressed populations were used:

1. Swarna-Sub1 pyramided lines with *qDTY*_*1.1*_*, qDTY*_*2.1*_, and *qDTY*_*3.1*_
2. IR64-Sub1 pyramided lines with *qDTY*_*1.1*_*, qDTY*_*1.2*_*, qDTY*_*2.2*_*, qDTY*_*2.3*_*, qDTY*_*3.2*_, and *qDTY*_*12.1*_
3. Samba Mahsuri pyramided lines with *qDTY*_*2.2*_ and *qDTY*_*4.1*_
4. TDK1-Sub1 pyramided lines with *qDTY*_*3.1,*_ *qDTY*_*6.1,*_ and *qDTY*_*6.2*_
5. MR219 pyramided lines with *qDTY*_*2.2*_*, qDTY*_*3.1,*_ and *qDTY*_*12.1*_

Three steps were employed for the development of a cost-effective, reliable, and resource-efficient combined MAS breeding strategy: (1) grain yield and genotypic data across F_3_, F_4_, F_5_, F_6_, F_7,_ and F_8_/fixed lines for all five sets were compiled; (2) class analysis was carried out to develop a combined MAS breeding strategy; and (3) the performance of the superior classes was monitored across advanced generations to validate the combined MAS breeding strategy.

The screening of all five population sets was carried out under NS control and RS drought stress conditions. For the NS experiments, 5-cm water depth level was maintained throughout the rice growing season until physiological maturity. For the screening under RS drought stress, irrigation was stopped at 30 days after transplanting (DAT). The last irrigation was provided at 24 DAT and there was no standing water in the field when drought was initiated at 30 DAT. The stress cycle was continued until severe stress symptoms were observed. Monitoring of soil water potential was carried out by placing perforated PVC pipes at 100-cm soil depth in the field in a zig-zag manner. After the initiation of stress, the water table level was recorded daily. When approximately 70% of the lines exhibited severe leaf rolling or wilting, one life-saving irrigation with a sprinkler system was provided. Then, a second cycle of the stress was initiated. The water table level was measured from all the pipes until the rice crop reached 50% maturity.

Molecular marker work was carried out following the procedure as described in Sandhu et al. (2014). For genotyping, a total of 754, 754, 432, 432, 432, and 52 plants were phenotyped and genotyped in F_3_ (NS, RS), F_4_ (NS), F_5_ (NS, RS), F_6_ (NS, RS), F_7_ (NS), and F_8_ (NS, RS) generations, respectively, in a Swarna-Sub1 background. In the IR64-Sub1 background, 467, 194, 64, 64, and 18 plants were phenotyped and genotyped in F_3_ (NS, RS), F_4_ (NS, RS), F_5_ (NS), F_6_ (NS, RS), and F_7_ (NS, RS) generations, respectively. In the Samba Mahsuri background, a total of 42, 3000, 1200, 70, 20 and 20 plants were phenotyped and genotyped in BC_1_F_3_ (NS, RS), BC_1_F_4_ (NS, RS), BC_1_F_5_ (NS), BC_1_F_6_ (NS), BC_1_F_7_ (NS, RS), and BC_1_F_8_ (NS, RS) generations respectively. In the TDK-1Sub1 background, 843, 231, 48, 48, 60 and 60 plants were phenotyped and genotyped in BC_2_F_3_ (RS), BC_2_F_4_ (NS, RS), BC_2_F_5_ (NS), BC_2_F_6_ (NS, RS), BC_2_F_7_ (NS, RS), and BC_2_F_8_ (NS, RS) generations, respectively. A total of 214, 620, 620, 70, and 70 plants were phenotyped and genotyped in BC_1_F_3_ (NS, RS), BC_1_F_4_ (NS), BC_1_F_5_ (NS, RS), BC_1_F_6_ (NS, RS), and BC_1_F_7_ (NS, RS) generations, respectively, in the MR219 background. Data on plant height, days to 50% flowering, and grain yield were recorded following the procedure of Venuprasad et al. (2009). The detailed description on QTLs and markers used in the present study in each background is presented in Additional file 1: Table S1. The general schematic scheme followed for QTL introgression and pyramiding program, phenotyping and genotyping screening is shown in Additional file 1: Figure S1.

### Analytical approach to reveal a combined MAS breeding strategy

The grain yield data from F_3_, F_4_, F_5_, F_6_, F_7_, and F_8_ generations across seasons and NS (control) and RS drought stress conditions for all five sets of pyramided populations were compiled and categorized into classes based on the genotypic QTL information. Class analysis using SAS v9.2 was attempted to see the mean grain yield performance of QTL classes across generation advancement.

### Genotyping and phenotyping cost calculation

The phenotyping cost of USD 36.18 per entry (two replications, screening under NS and RS drought stress with plot size of 1.54 m^2^) (IRRI Standard drought screening costing) including the cost of land preparation, land rental, irrigation, electricity, field layout, seeding, transplanting, maintenance cost, resource input (fertilizer), pesticides, herbicides, field supplies, harvesting, threshing, drying, data collection, and labor was used to calculate the cost savings for phenotyping. The genotyping cost was calculated for the whole population across successive generations (F_3_ to F_7_/F_8_) and compared with the genotyping cost (F_3_ to F_7_/F_8_) considering only the QTL classes that performed better in F_3_. The genotyping cost was calculated considering five markers per QTL (one peak/near the peak, two right-hand-side flanking markers, and two left-hand-side flanking markers) using USD 0.50 per data point (Xu et al. 2002; Xu 2010).

### Statistical analysis

#### Mean comparison of QTL genotype classes

Hypothesis about no differences among phenotype means of QTL genotype classes for each background under NS and RS drought stress in each season was performed in SAS v9.2 (SAS Institute Inc. 2009) using the following linear model.

$$ {y}_{ij kl}=\mu +{r}_k+b{(r)}_{kl}+{q}_i+g{(q)}_{ij}+{e}_{ij kl} $$

where *μ* represents the population mean, *r*_*k*_ represents the effect of the *k*^*th*^ replicate, *b(r)*_*kl*_ is the effect of the *l*^*th*^ block within the *k*^*th*^ replicate, *q*_*i*_ corresponds to the effect of the *i*^*th*^ QTL, *g(q)*_*ij*_ symbolizes the effect of the *j*^*th*^ genotype nested within the *i*^*th*^ QTL, and *e*_*ijkl*_ corresponds to the error (Knapp 2002). The effects of QTL class and the genotypes within QTL were considered fixed and the replicates and blocks within replicates were set to random.

## Additional file

Additional file 1: **Table S1.** QTLs and markers information’s in marker assisted introgression program in different backgrounds. **Figure S1.** General schematic scheme for QTL introgression and pyramiding program, phenotyping and genotyping screening. In case of Swarna-Sub1 and IR64-Sub1 no backcross was attempted. In case of Samba Mahsuri and MR219, one backcross was attempted. In case of TDK1-Sub1 two backcross was attempted. (DOCX 269 kb)
