# Supplementary material for: Marker-assisted selection strategy to pyramid two or more QTLs for quantitative trait-grain yield under drought
Source: Rice (N Y). 2018 May 29;11:35. doi: 10.1186/s12284-018-0227-0 (PMC5975061; doi:10.1186/s12284-018-0227-0)
Supplement: Supplementary file 1 — Table S1. QTLs and markers information’s in marker assisted introgression program in different backgrounds. Figure S1. General schematic scheme for QTL introgression and pyramiding program, phenotyping and genotyping screening. In case of Swarna-Sub1 and IR64-Sub1 no backcross was attempted. In case of Samba Mahsuri and MR219, one backcross was attempted. In case of TDK1-Sub1 two backcross was attempted. (DOCX 269 kb) [file 12284_2018_227_MOESM1_ESM.docx]

| **Background** | **QTLs** | **Marker** |
| --- | --- | --- |
| Swarna-Sub1 | *qDTY_1.1_* | RM11943, RM12023, RM12091, RM12233 |
|  | *qDTY_2.1_* | RM5791, RM521, RM3549,RM324, RM6374 |
|  | *qDTY_3.1_* | RM416, RM16030, RM520 |
| IR64-Sub1 | *qDTY_1.1_* | RM11943, RM12023, RM12233 |
|  | *qDTY_1.2_* | RM212, RM3825, RM315 |
|  | *qDTY_2.2_* | RM236, RM279, RM555 |
|  | *qDTY_12.1_* | RM28048, RM28130,RM28099, CG29430, indel8 |
|  | *qDTY_2.3_* | RM3212, RM573, RM1367 |
|  | *qDTY_3.1_* | RM523, RM22, RM545 |
|  | *qDTY_4.1_* | RM518, RM335, RM16368 |
| Samba Mahsuri | *qDTY_2.2_* | RM236, RM279, RM555 |
|  | *qDTY_4.1_* | RM518, RM335, RM16368 |
| TDK1-Sub1 | *qDTY_3.1_* | RM55, RM168, RM186,RM293, RM468 |
|  | *qDTY_6.1_* | RM204, RM217, RM508,RM586, RM587 |
|  | *qDTY_6.2_* | RM3, RM541 |
| MR219 | *qDTY_2.2_* | RM236, RM279, RM12460 |
|  | *qDTY_3.1_* | RM416, RM16030, RM520 |
|  | *qDTY_12.1_* | RM28048, RM511, RM28099,RM28166, CG29430, indel8, RM28130 |

Table S1 QTLs and markers information’s in marker assisted introgression program in different backgrounds.

*Source: Sandhu and Kumar 2017*


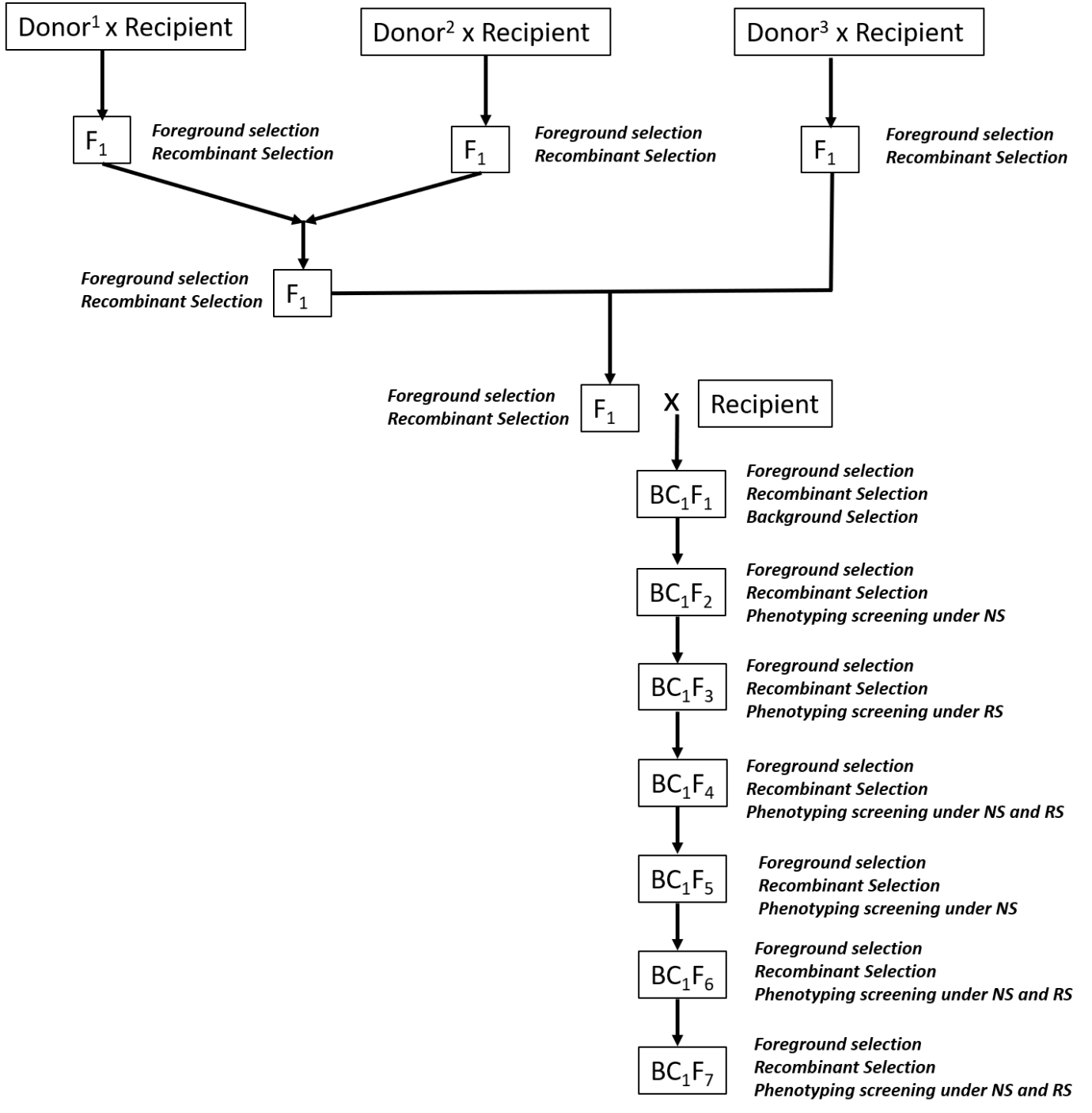


Figure S1 General schematic scheme for QTL introgression and pyramiding program, phenotyping and genotyping screening. In case of Swarna-Sub1 and IR64-Sub1 no backcross was attempted. In case of Samba Mahsuri and MR219, one backcross was attempted. In case of TDK1-Sub1 two backcross was attempted.
